# Manipulating Ratio Spectra for the Spectrophotometric Analysis of Diclofenac Sodium and Pantoprazole Sodium in Laboratory Mixtures and Tablet Formulation

**DOI:** 10.1155/2014/495739

**Published:** 2014-02-18

**Authors:** Nejal M. Bhatt, Vijay D. Chavada, Mallika Sanyal, Pranav S. Shrivastav

**Affiliations:** ^1^Department of Chemistry, School of Sciences, Gujarat University, Ahmedabad, Gujarat 380009, Gujarat, India; ^2^Department of Chemistry, St. Xavier's College, Navrangpura, Ahmedabad, Gujarat 380009, Gujarat, India

## Abstract

*Objective*. Three sensitive, selective, and precise spectrophotometric methods based on manipulation of ratio spectra, have been developed and validated for the determination of diclofenac sodium and pantoprazole sodium. *Materials and Methods*. The first method is based on ratio spectra peak to peak measurement using the amplitudes at 251 and 318 nm; the second method involves the first derivative of the ratio spectra (Δ*λ* = 4 nm) using the peak amplitudes at 326.0 nm for diclofenac sodium and 337.0 nm for pantoprazole sodium. The third is the method of mean centering of ratio spectra using the values at 318.0 nm for both the analytes. *Results*. All the three methods were linear over the concentration range of 2.0–24.0 **μ**g/mL for diclofenac sodium and 2.0–20.0 **μ**g/mL for pantoprazole sodium. The methods were validated according to the ICH guidelines and accuracy, precision, repeatability, and robustness are found to be within the acceptable limit. The results of single factor ANOVA analysis indicated that there is no significant difference among the developed methods. *Conclusions*. The developed methods provided simple resolution of this binary combination from laboratory mixtures and pharmaceutical preparations and can be conveniently adopted for routine quality control analysis.

## 1. Introduction

**Figure 1:**
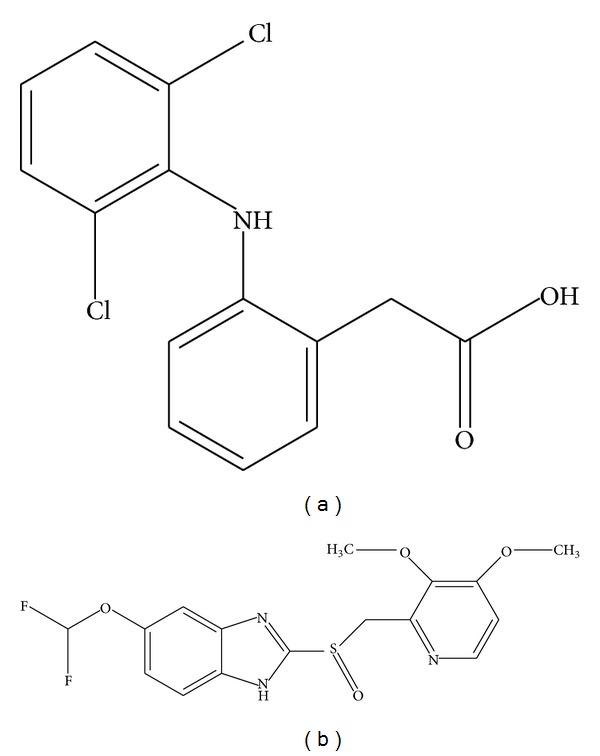
Chemical structures of (a) diclofenac and (b) pantoprazole.

Nonsteroidal anti-inflammatory drugs (NSAIDs) are a class of drugs that provide analgesic, antipyretic, and anti-inflammatory effects [1]. Diclofenac (DCL, Figure 1(a)) is one of the most prescribed NSAIDs worldwide, used to reduce inflammation and as an analgesic to reduce pain in certain other conditions [2]. The primary mechanism responsible for anti-inflammatory, antipyretic, and analgesic action of DCL is thought to be inhibition of prostaglandin synthesis by inhibition of cyclooxygenase (COX) [3]. Inhibition of COX also decreases prostaglandins in the epithelium of the stomach, making it more sensitive to corrosion by gastric acid. The threats of peptic ulcer on long-term use of DCL require concomitant treatment with proton pump inhibitors as they have been shown to be effective in preventing the development of gastric and duodenal ulcers in high-risk patients taking NSAIDs [4]. Pantoprazole (PAN, Figure 1(b)) is a type of medicine called a proton pump inhibitor (PPI). PAN suppresses the final step in gastric acid production by covalently binding to the (H+, K+)-ATPase enzyme system at the secretory surface of the gastric parietal cell. This effect leads to inhibition of both basal and stimulated gastric acid secretion, irrespective of the stimulus. Besides inhibiting acid secretion, PAN affords protection against NSAID-induced gastric damage [5], has low potential for drug-drug interactions, and is particularly suitable for administration to elderly patients who often require concomitant treatment with NSAIDs as no dose adjustment is required during concomitant treatment with both drugs [4].

Literature survey revealed several analytical methods reported for DCL estimation using HPLC [6–10] and electroanalytical techniques [11–14]. Aurora-Prado et al. [15] have developed a capillary electrophoresis method for determination of DCL in tablets and compared its performance with that of an HPLC method. Several spectrophotometric methods have been reported based on either conventional mathematical resolution or using chemometric approach as reviewed by Gouda et al. [16]. Besides, some methods based on planar chromatography are also described for determination of DCL from pharmaceutical preparations [17–20]. On the other hand, many chromatographic methods presented for the determination of PAN either alone or in combination with other drugs have been reported using HPLC [21–24] and HPTLC [25–28]. Some nonchromatographic methods for PAN may include methods based on electrophoresis [29], electroanalytical techniques [30, 31], and spectrophotometry [32–34]. However, to the best of our knowledge, there are no methods for the simultaneous quantitation of DCL and PAN. In addition, ratio manipulating methods have potential application in pharmaceutical analysis as they provide simple and effective results for binary mixtures compared to conventional spectrophotometric methods [35–39].

In the present work, three different methods based on manipulation of ratio spectra are described for the simultaneous determination of DCL and PAN. These methods demonstrate a simple and accurate approach for the analysis of this binary mixture without the need of sophisticated instruments and expensive solvents. All the methods give reliable and precise results for their analysis in bulk drug, laboratory mixtures, and one tablet formulation.

## 2. Materials and Methods

### 2.1. Chemicals and Materials

Reference standard of diclofenac sodium (99.48%) was procured from Titan Pharmaceuticals Ltd. (Mumbai, India), while pantoprazole sodium (99.58%) was obtained from Asutosh Pellets (Gujarat, India). Methanol used was of spectroscopic grade purchased from E. Merck (Mumbai, India). Deionized water was prepared from Milli-Q water purification system purchased from Millipore (Bangalore, India). Ten tablets of Dufex from CFL Pharmaceuticals Ltd. (Mumbai, India) labeled to contain 75 mg of DCL and 20 mg of PAN were purchased from local pharmacy.

### 2.2. Instrumentation and Analysis Conditions

A Shimadzu UV-1700 double beam spectrophotometer (Kyoto, Japan) with matched 10 mm quartz cells was used for spectral measurements. The parameters used were wavelength accuracy: ±0.5 nm, bandwidth: 1 nm, and scan speed: 400 nm/min. Spectral data were processed with Shimadzu UV PC software version 2.0. Sartorius GD503 (Bradford, MA, USA) analytical balance, having a readability of 0.0001 g, was used for weighing samples throughout the study. Matlab Version 6.5 was used for mean centering of ratio spectra method.

### 2.3. Solution Preparation

The standard stock solutions of the studied drugs having 200 *μ*g/mL concentrations were prepared by dissolving requisite amount of each drug in 20 mL methanol and then diluted to 100 mL with the same diluent. The standard solutions were stored in a refrigerator (below 4°C) and were found to be stable for a minimum period of 2 weeks.

### 2.4. Spectral Characteristics of DCL and PAN

The zero-order absorption spectrum for 8.0 *μ*g/mL of DCL and 8.0 *μ*g/mL of PAN were recorded against methanol as a blank over 200–400 nm wavelength range.

### 2.5. Construction of Calibration Curves

Calibrators in the concentration range of 2.0–24.0 *μ*g/mL for DCL and 2.0–20.0 *μ*g/mL for PAN were prepared from standard stock solutions (200 *μ*g/mL) in two separate series of 10 mL volumetric flasks in methanol. The spectra of these standard solutions were recorded from 200 to 400 nm wavelength range.

### 2.6. Method 1: Ratio Spectra Peak to Peak Measurement (RPP)

For the determination of DCL in presence of PAN, the spectra of DCL were divided by the spectrum of 4.0 *μ*g/mL PAN, smoothed with Δ*λ* = 16 nm. The peak to peak amplitudes in the obtained DCL ratio spectra between 251 and 318 nm were measured and plotted versus the corresponding concentration. For the determination of PAN in presence of DCL, the spectra of PAN were divided by the spectra of 4.0 *μ*g/mL DCL. The peak to peak amplitudes in the PAN ratio spectra were measured between 318 and 251 nm in a similar way. A calibration graph relating to amplitude difference with corresponding concentrations in *μ*g/mL of PAN was constructed.

### 2.7. Method 2: Ratio Derivative (^1^DD)

For the determination of DCL in presence of PAN, the spectra of DCL were divided by the spectrum of 4.0 *μ*g/mL PAN, smoothed with Δ*λ* = 16 nm, followed by derivatization of the ratio spectra with Δ*λ* = 4 nm. A calibration graph relating the peak amplitude at 326.0 nm to the corresponding concentrations of DCL (*μ*g/mL) is constructed. Similarly, for the determination of PAN in presence of DCL, the spectra of PAN were divided by the spectrum of 4.0 *μ*g/mL DCL, and then the first derivative of the ratio spectra (^1^DD) with Δ*λ* = 4 nm was obtained. A calibration graph is constructed by plotting the peak amplitude at 337.0 nm to the corresponding PAN concentration (*μ*g/mL).

### 2.8. Method 3: Mean Centering of the Ratio Spectra (MCR)

The scanned spectra of DCL were divided by the spectrum of 4.0 *μ*g/mL PAN and the obtained ratio spectra are smoothed with Δ*λ* = 16 nm and then mean centered. A similar treatment was applied to PAN spectra by dividing with the spectra of 4.0 *μ*g/mL DCL, followed by mean centering. The calibration curves for both DCL and PAN were constructed by plotting the mean centered values at 318.0 nm for both DCL and PAN, versus the corresponding concentration.

### 2.9. Determination of DCL and PAN in Laboratory-Prepared Mixtures

Aliquots equivalent to 80, 80, 80, 100, 100, 100, 160, and 160 *μ*g of DCL were transferred from its standard working solution into a series of 10 mL measuring flasks. To the same flasks, aliquots equivalent to 80, 100, 160, 80, 100, 160, 80, and 100 *μ*g of standard working solution of PAN were added, completed to volume with methanol, and mixed well.

### 2.10. Determination of DCL and PAN in Pharmaceutical Tablets

Ten tablets of Dufex (claimed values: 75.0 mg DCL and 20.0 mg PAN) were accurately weighed and finely powdered. An amount of the powder equivalent to 15 mg of DCL and 2.0 mg PAN was taken and dissolved in about 50 mL methanol by shaking in ultrasonic bath for about 5 min. The solutions are filtered and transferred quantitatively into two separate 100 mL volumetric flasks. The volume was then completed to the mark with methanol. Working solutions were prepared by appropriate dilution of the stock solutions with methanol.

## 3. Results

### 3.1. Optimization of Conditions for Ratio Spectra

**Figure 2:**
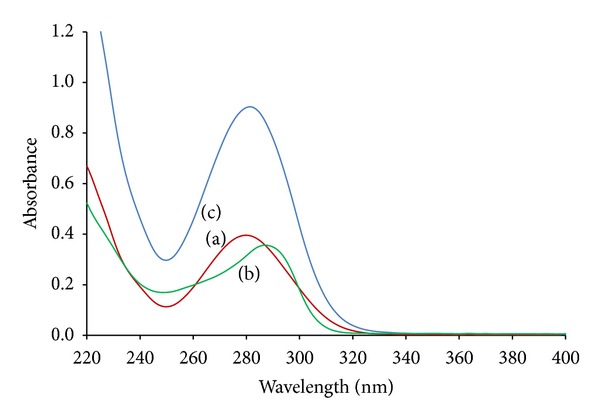
Overlay zero-order spectra of (a) diclofenac, (b) pantoprazole, and (c) pharmaceutical tablet formulation.

Molecular absorption spectroscopy has been extensively used for the determination of drugs in pharmaceutical preparations with a view to develop simple, rapid, and reliable analytical methods. The use of this technique for pharmaceutical analyses has the inherent constraint that most active drugs absorb in the UV region and exhibit strongly overlapped spectra that impede their simultaneous determination. The zero-order absorption spectra of DCL and PAN showed extensive overlapping (Figure 2), which prevented the direct resolution of both analytes. In such cases, spectrophotometric methods based on manipulation of ratio spectra can be used for their simultaneous determination.

During method development, the main parameters which affect the shape of the ratio spectra which are the wavelength, scanning speed, the concentration of the standard solution used as a divisor, and the wavelength increment to derive the derivative (Δ*λ*) were carefully optimized. It was found that higher scanning speed resulted in noisy spectra; on the other hand, at low scanning speed the noise was reduced but with increased time for measurement. Hence, an optimum scanning speed of 400 nm/min was selected for balanced outcome. A study was carried out to examine the effect of divisor concentration on the ratio spectra of DCL and PAN. The divisor concentration was tested in the range of 2.0–8.0 *μ*g/mL. With an increase or decrease in divisor concentration, there was a corresponding decrease or increase in the resulting absorbance ratio values, respectively. However, the positions of the peaks and troughs remain unaffected. Divisor concentration of 4.0 *μ*g/mL for either DCL or PAN produced the best results in terms of accuracy, repeatability, and recovery in laboratory prepared mixtures and tablet formulation.

### 3.2. Ratio Spectra Peak to Peak Measurement [36]

For a mixture of X and Y, the following equation can be formed if there is no interaction among two components and the Beer's law is obeyed for each component:

(1) $$\begin{array}{c} A_{\text{M}}=\alpha_{\text{X}}C_{\text{X}}+\alpha_{\text{Y}}C_{\text{Y}}, \end{array}$$

where *A*_M_ is the absorbance of the mixture, *α*_X_ and *α*_Y_ are the molar absorptivities of X and Y, and *C*_X_ and *C*_Y_ are the concentrations of X and Y, respectively. On dividing (1) with the absorbance of standard solution of pure component Y (*A*_Y°_ = *α*_Y°_ *C*_Y°_), the following equation results:

(2) $$\begin{array}{c} \frac{A_{\text{M}}}{A_{{\text{Y}}^{{}^{\circ}}}}=\frac{A_{\text{X}}}{A_{{\text{Y}}^{{}^{\circ}}}}+\frac{C_{\text{Y}}}{C_{{\text{Y}}^{{}^{\circ}}}}. \end{array}$$

The term *C*_Y_/*C*_Y°_ is constant, which can be eliminated when the difference in absorbance ratio amplitudes between two wavelengths (peak to peak) is considered:

(3) $$\begin{array}{c} {\left(\frac{A_{\text{M}}}{A_{{\text{Y}}^{{}^{\circ}}}}\right)}_{\lambda 1}-{\left(\frac{A_{\text{M}}}{A_{{\text{Y}}^{{}^{\circ}}}}\right)}_{\lambda 2}={\left(\frac{A_{\text{X}}}{A_{{\text{Y}}^{{}^{\circ}}}}\right)}_{\lambda 1}-{\left(\frac{A_{\text{X}}}{A_{{\text{Y}}^{{}^{\circ}}}}\right)}_{\lambda 2}. \end{array}$$

Equation (3) indicates that the difference in amplitude at two wavelengths (called as *peak to peak* or *peak to trough* measurement) is proportional to the concentration of component X, and the amplitude difference obtained for pure compound X remains the same when analyzed with different amounts of compound Y, as the interference of the constant cancels out. To find the amount of X, a calibration curve may be plotted by using the difference in amplitude obtained through analyzing pure standard solutions of X. Similar treatment may be developed for the quantitation of another component Y.

**Figure 3:**
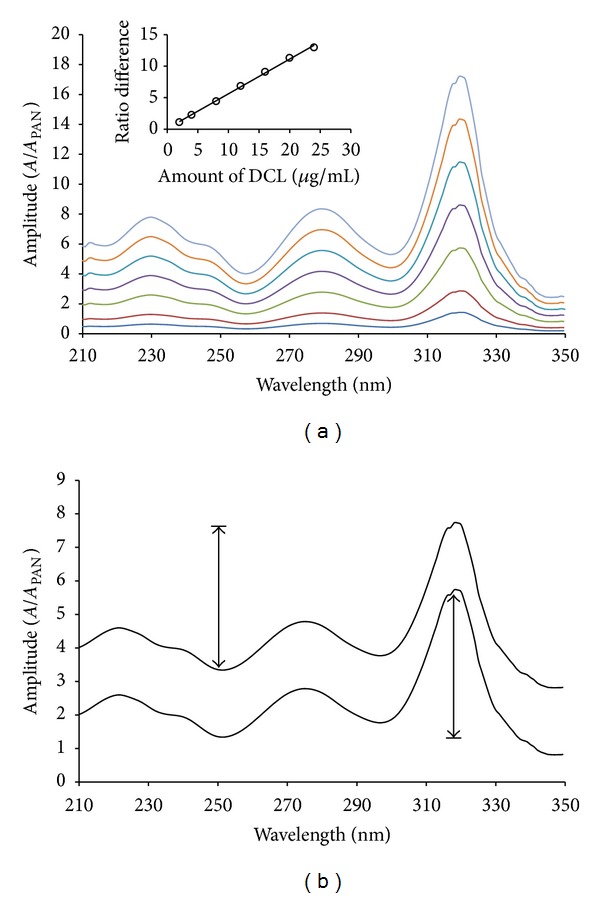
(a) Division spectra of 2.0–24.0 *μ*g/mL diclofenac and (b) division spectra of a standard solution and a mixture solution containing the same concentration of diclofenac, using 4.0 *μ*g/mL pantoprazole as a divisor and methanol as a solvent.

**Figure 4:**
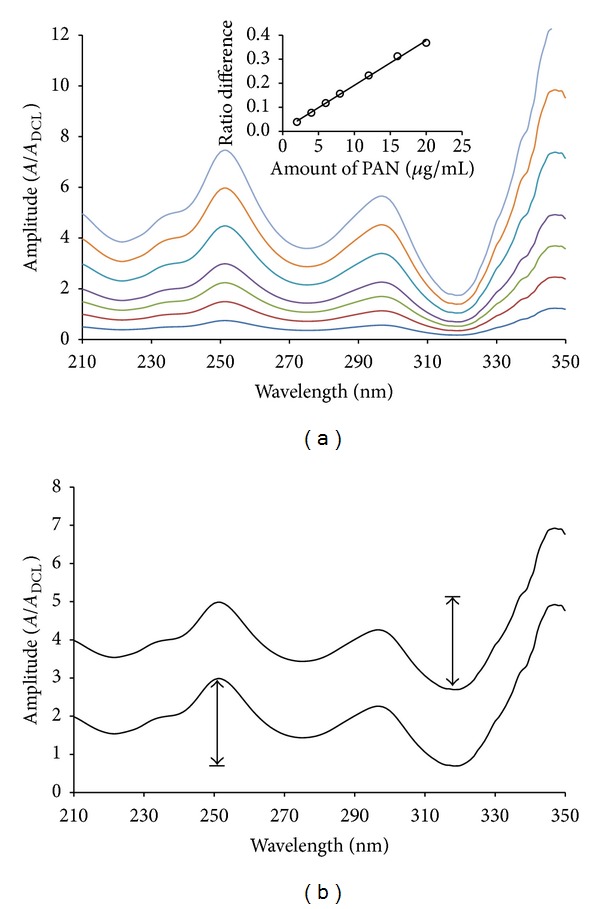
(a) Division spectra of 2.0–20.0 *μ*g/mL pantoprazole and (b) division spectra of a standard solution and a mixture solution containing the same concentration of pantoprazole, using 4.0 *μ*g/mL diclofenac as a divisor and methanol as a solvent.

The ratio spectra of different DCL standards with increasing concentrations in methanol, obtained by dividing each by the spectrum of 4 *μ*g/mL PAN in the same solvent, are illustrated in Figure 3(a). The peak to trough amplitudes between 318.0 nm (peak) and 251.0 nm (trough) on the generated ratio spectra are proportional to DCL concentration.  Figure 3(b) shows the ratio spectra of a standard solution of DCL and a mixture solution containing the same concentration of DCL. The difference between the two spectra gives the constant “interference” value (the term *C*_Y_/*C*_Y°_ in (2)). This constant interference can be eliminated by measurement of the absorbance ratio difference between two selected wavelengths 318.0 nm (peak) and 251.0 nm (trough). Ideally, these selected wavelengths should correspond to the peak and the trough in the ratio spectrum in order to achieve the highest sensitivity.  Figure 3(b) shows that the peak to trough amplitude in the mixture spectrum is equal to that in standard DCL spectrum; therefore, DCL can be determined in the mixture without interference from PAN. For the determination of PAN, an analogous procedure was followed. Contrary to DCL, the ratio spectra of different standard solutions of PAN using 4 *μ*g/mL DCL as divisor show the peak and trough at 251.0 and 318.0 nm, respectively (Figure 4(a)). The peak to trough amplitudes on the ratio spectra were measured and found proportional to PAN concentration. From Figure 4(b), it is evident that the measured peak to trough amplitude in the mixture spectrum is equal to that in standard PAN spectrum irrespective of the DCL concentration.

### 3.3. Ratio Derivative Method

This simple spectrophotometric method, developed by Salinas et al. [37], is based on the derivation of the ratio spectra for resolving binary mixtures. It permits the use of the wavelength of highest value of analytical signals with several maxima and minima, which give an opportunity for the determination of active compounds in the presence of other compounds and excipients which could possibly interfere in the assay. In this method, the absorption spectrum of the mixture (absorbance at each wavelength) is divided by the absorption spectrum of a standard solution of one of the components, and the first derivative of the ratio spectrum is obtained. The concentration of the other components is then determined from a calibration graph.

**Figure 5:**
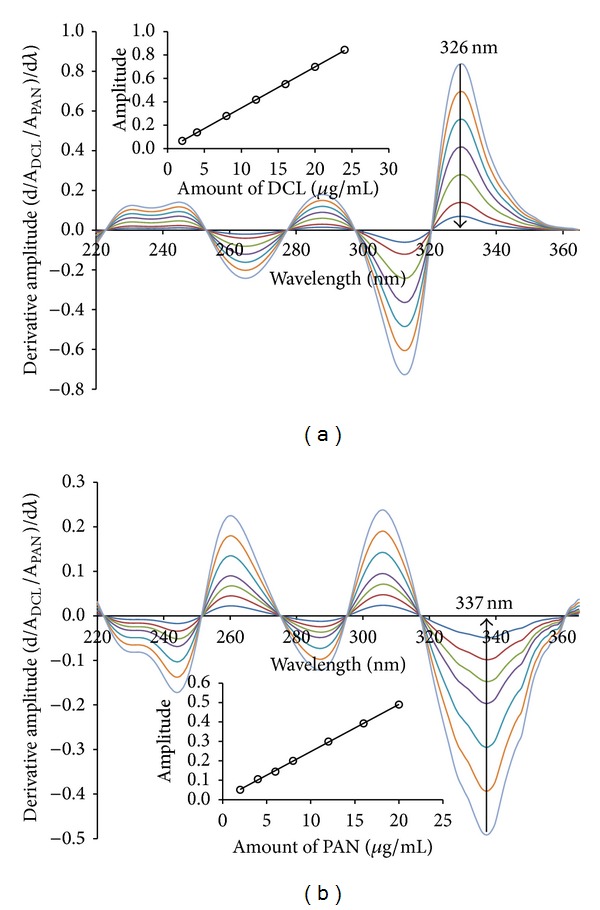
First derivative of ratio spectra of (a) 2.0–24.0 *μ*g/mL diclofenac and (b) 2.0–20.0 *μ*g/mL pantoprazole obtained using 4.0 *μ*g/mL pantoprazole and diclofenac as a divisor, respectively, and methanol as a solvent.

The difference between the two spectra (Figures 3(b) and 4(b)), that is, the constant interference value due to compound Y (the term *C*_Y_/*C*_Y°_ in (2)), was eliminated in Salinas method [37] by recording the first derivative of the ratio spectrum. As seen in the derivative ratio spectra of DCL (Figure 5(a)), there exist two maxima (326 and 286.0 nm) and two minima (310.0 and 263.0 nm). Good linearity was observed at 326.0 and 310.0 nm, but the recovery percent and precision at 326.0 nm was better which may be attributed to its higher signal to noise ratio. For determination of PAN, measurements at 260, 306, and 337 nm (Figure 5(b)) showed comparable linearity, but good recovery was observed at 337.0 nm. Measuring the amplitude between 306.0 and 337.0 nm did not show significant improvement in the recovery percent. The peak amplitudes of the first derivative of ratio spectra were then recorded at 326.0 nm for DCL and 337.0 nm for PAN. Under the established conditions, good linearity was obtained in the concentration range of 2.0–24.0 *μ*g/mL and 2.0–20.0 *μ*g/mL for both DCL and PAN, respectively. The linear regression equations were found to be

(4) $$\begin{array}{c} P_{\text{DCL}}=0.0351C_{\text{DCL}}-0.0045\;\;r^{2}=0.9998,\\ P_{\text{PAN}}=0.0243C_{\text{PAN}}+0.0039\;\;r^{2}=0.9996, \end{array}$$

where *C*_DCL_ and *C*_PAN_ are the concentration (*μ*g/mL) of DCL and PAN, respectively, *P*_DCL_ and *P*_PAN_ are the peak amplitude of the first derivative of the ratio spectrum for DCL and PAN, respectively, and *r* ^2^ is the correlation coefficient.

### 3.4. Mean Centering of Ratio Spectra

For further improvement of the selectivity to resolve the overlap present between DCL and PAN, a simple method is applied based on the mean centering of ratio spectra [38]. This eliminates the derivative step and therefore the signal-to-noise ratio is enhanced.

If there is no interaction among two components of a mixture, that is, X and Y, and if Beer's law is obeyed for each compound, it can be expressed as follows:

(5) $$\begin{array}{c} A_{\text{M}}=\alpha_{\text{X}}C_{\text{X}}+\alpha_{\text{Y}}C_{\text{Y}}, \end{array}$$

where *A*_M_ is the absorbance of the mixture, *α*_X_ and *α*_Y_ are the molar absorptivities of X and Y, and *C*_X_ and *C*_Y_ are the concentrations of X and Y, respectively.

If (5) is divided by *α*_Y_, the ratio spectrum is obtained in the form of the following equation:

(6) $$\begin{array}{c} B=\frac{A_{\text{M}}}{\alpha_{\text{Y}}}=\frac{\alpha_{\text{X}}C_{\text{X}}}{\alpha_{\text{Y}}}+C_{\text{Y}}. \end{array}$$

Since the mean centering of a constant (*C*_Y_) is zero, mean centering (MC) of (6) would be obtained as

(7) $$\begin{array}{c} \text{MC}\left(B\right)=\text{MC}\left[\frac{\alpha_{\text{X}}C_{\text{X}}}{\alpha_{\text{Y}}}\right]. \end{array}$$

Equation (7) illustrates the mathematical explanation for analysis of binary components that permits the determination of concentration of one compound without interference from the other compound of the binary system, and vice versa. According to this equation, there is a linear relation between the amount of MC(*B*) and the concentration of X in the solution, which can be demonstrated by plotting a calibration curve for MC(*B*) against concentration of particular analyte in the standard solutions of that analyte or in the standard binary mixtures. In addition, measurement of MC(*B*) values corresponding to maxima or minima provides improvement in the sensitivity. Similar procedure may be performed for Y as described for X.

**Figure 6:**
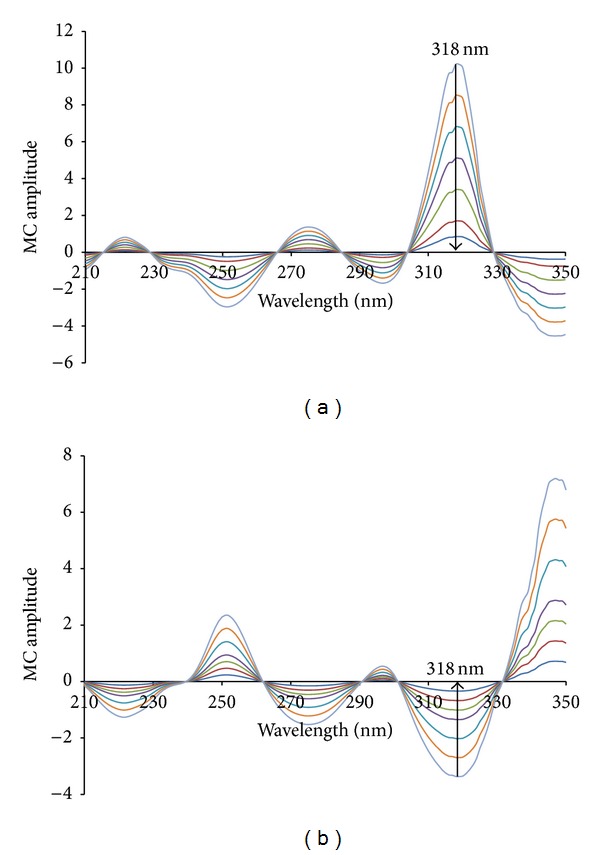
The mean centered ratio spectra of (a) 2.0–24.0 *μ*g/mL diclofenac and (b) 2.0–20.0 *μ*g/mL pantoprazole obtained using 4.0 *μ*g/mL pantoprazole and diclofenac as a divisor, respectively, and methanol as a solvent.

MCR was applied for the quantitation of DCL and PAN in their laboratory prepared mixtures and pharmaceutical preparations. First, the absorption spectra of DCL were divided by the absorption spectrum of 4 *μ*g/mL PAN and the absorption spectra of PAN are divided by the absorption spectrum of 4 *μ*g/mL DCL for determination of DCL and PAN, respectively (Figures 3(a) and 4(a)). Then, the ratio spectra were mean centered using Matlab (version 6.5) as presented in Figure 6(a), which showed a maxima at 318.0 nm and minima at 251.0 nm for DCL. The mean centered ratio spectra of PAN (Figure 6(b)) indicated the presence of two minima at 318 nm and 275 nm, respectively, and one maximum at 251 nm. After due considerations of sensitivity and recovery, the mean centered amplitudes at the same wavelength at 318 nm were selected for the measurement of DCL and PAN, respectively. The linear regression equations were

(8) $$\begin{array}{c} {\text{MC}}_{\text{DCL}}=0.4260C_{\text{DCL}}-0.0071\;\;r^{2}=0.9998,\\ {\text{MC}}_{\text{PAN}}=0.1668C_{\text{PAN}}+0.0125\;\;r^{2}=0.9991, \end{array}$$

where *C*_DCL_ and *C*_PAN_ are the concentrations (*μ*g/mL) of DCL and PAN, respectively, MC is the peak amplitude of the mean centered ratio spectrum curve, and *r* ^2^ is the correlation coefficient.

### 3.5. Method Validation [39]

#### 3.5.1. Linearity

The calibration range was established based on adherence to Beer's law and the concentration of DCL and PAN present in the pharmaceutical preparations to give accurate precise and linear results. The linearity of the methods was established by analyzing seven concentrations of DCL and PAN ranging between 2.0–24.0 *μ*g/mL and 2.0–20.0 *μ*g/mL, respectively. Each concentration was analyzed in triplicate. The assay was performed according to the established conditions and the results are as summarized in Table 1.

#### 3.5.2. Limits of Detection and Quantification

Limits of detection (LOD) and limit of quantification (LOQ) were calculated according to the ICH guidelines [33]. LOD was defined as 3.3*S/b* and LOQ was computed as 10*S/b*, where *S* is the standard deviation of the response and *b* is the slope of the calibration curve. Standard deviation was calculated by replicate analysis of pure standard solutions of 4 *μ*g/mL DCL and PAN. The sensitivity of the proposed method can be confirmed by the low LOD and LOQ values as shown in Table 1.

#### 3.5.3. Accuracy and Precision

In order to assess the intraday precision and accuracy, for the proposed methods, three replicate determinations at three different concentration levels were carried out in the same day. The interday precision and accuracy were assessed similarly for three replicate determinations of the same concentration levels on three consecutive days. The concentrations were calculated using the corresponding regression equations. The precision values expressed as % RSD were less than 2%, while the accuracy ranged from 99.25 to 101.05% as shown in Table 2.

#### 3.5.4. Selectivity

The selectivity of the proposed procedures is assessed by the analysis of laboratory prepared mixtures containing different ratios of the two drugs, where satisfactory results are obtained over the calibration ranges as shown in Table 3. The proposed procedures are also applied for the determination of DCL and PAN in Dufex tablets. The validity of the proposed procedures is further assessed by applying the standard addition technique. The results obtained are given in Table 4.

#### 3.5.5. Stability of Solutions

DCL and PAN stock and calibration solutions remained unaffected for a minimum period of 2 weeks when stored at 4°C. There was practically no change in the concentration as evident from spectrophotometric measurements.

### 3.6. Statistical Comparison

One-way ANOVA was applied for the purpose of comparison of developed spectrophotometric methods.  Table 5 shows that there was no significant difference between these ratio manipulating methods when they were applied for the determination of DCL and PAN in the pharmaceutical formulation.

### 3.7. Determination of DCL and PAN in Laboratory-Prepared Mixtures

The standard mixtures containing different ratios of each drug (above and below their normal ratios in tablets) were prepared and analyzed by the developed methods. The recovered drug concentration values for DCL and PAN in mixtures (Table 3) demonstrate the analytical power of ratio methods to resolve and quantify the investigated drugs when present in different proportions.

### 3.8. Determination of DCL and PAN in Pharmaceutical Tablets

The proposed methods were successfully applied to the analysis of both drugs in their pharmaceutical preparations. Recoveries were calculated using regression analysis and standard addition methods. The results obtained were precise and in good agreement with the labeled claim as apparent from the satisfactory values of recovery and % RSD shown in Table 4. Further, the recovery studies indicate that all the methods are practically free from interference due to tablets additives.

## 4. Conclusion

In summary, three spectrophotometric methods, based on manipulation of ratio spectra, were developed and validated for the analysis of diclofenac and pantoprazole in binary mixtures following the ICH guidelines. This is the first report on the simultaneous quantitation of this drug combination with high clinical relevance. The developed methods are simple, precise, and sensitive. The ratio derivative method was able to determine both the components of the binary mixture; however, the procedure is comprised of three steps, which included a division step followed by derivatization and construction of calibration curve. Due to the fact that the derivatization decreases the sensitivity, ^1^DD is less adequate for analysis; nevertheless, it offers several maxima and minima in the derivative ratio spectra which help in the determination of active components in the presence excipients. On the other hand, ratio spectra *peak to peak* measurement method was able to determine both drugs using the simple two-step procedure, that is, a division and measurement of difference between the peak and trough. In addition, it does not require specific software for complex mathematical treatment of the data which is a prerequisite for mean centering of ratio spectra. However, MCR provides better resolution of the components using automated manipulation of the spectral data. Further, this approach helps to minimize mutual interference between the drugs compared to the other two methods. In addition, it does not require any derivatization step which increases the signal to noise ratio. Finally, based on the overall performance of these methods, they can be readily applied in quality control laboratories which do not have sophisticated instruments like HPLC.

## Figures and Tables

**Table 1:** Regression parameters obtained for the determination of diclofenac (DCL) and pantoprazole (PAN) using the proposed methods.

Parameters	Ratio spectra peak to peak measurement		Ratio derivative method		Mean centering of ratio spectra	
	DCL	PAN	DCL	PAN	DCL	PAN
Wavelength(s) (in nm)	318.0 and 251.0	318.0 and 251.0	326.0	337.0	318.0	318.0
Calibration range (*μ*g/mL)	2.0–24.0	2.0–20.0	2.0–24.0	2.0–20.0	2.0–24.0	2.0–20.0
Slope	0.5507	0.0187	0.0351	0.0243	0.4260	0.1668
Intercept	0.1112	0.0045	−0.0048	0.0039	−0.0071	0.0125
Correlation coefficient (*r* ^2^)	0.9981	0.9977	0.9998	0.9996	0.9998	0.9991
LOD (*μ*g/mL)	0.583	0.597	0.605	0.688	0.560	0.543
LOQ (*μ*g/mL)	1.765	1.811	1.832	2.085	1.696	1.645

LOD: limit of detection; LOQ: limit of quantitation.

**Table 2:** Accuracy and precision results of diclofenac and pantoprazole in bulk form using the proposed methods.

Amount added (µg/mL)	Intrabatch^a^						Interbatch^b^					
	RPP		^ 1^DD		MCR		RPP		^ 1^DD		MCR	
	Recovery (%)	% RSD	Recovery (%)	% RSD	Recovery (%)	% RSD	Recovery (%)	% RSD	Recovery (%)	% RSD	Recovery (%)	% RSD
Diclofenac												
12.0	100.18	0.637	100.58	0.708	100.28	0.676	99.25	0.808	99.67	0.893	100.61	0.625
15.0	99.62	0.697	100.42	0.894	100.31	0.655	99.41	0.778	100.18	0.468	99.43	0.618
18.0	100.29	0.743	99.33	1.006	99.59	0.826	99.67	0.953	100.29	0.935	99.75	0.965

Pantoprazole												
3.2	99.83	0.599	100.04	0.823	100.34	0.763	99.29	0.928	100.24	0.815	99.75	0.664
4.0	100.60	0.879	99.43	0.702	100.17	0.739	101.05	0.933	100.49	0.789	100.31	0.750
4.8	100.16	0.708	100.25	0.910	99.91	0.631	99.65	0.808	99.69	0.895	100.23	0.820

^a^Samples analyzed in three replicates in the same day; ^b^samples analyzed in three replicates in three consecutive days; RPP: ratio spectra *peak to peak* measurement method; ^1^DD: ratio derivative method; MCR: mean centering of ratio spectra method; % RSD: percent relative standard deviation.

**Table 3:** Summary for the determination of diclofenac (DCL) and pantoprazole (PAN) in laboratory prepared mixtures by the proposed methods.

Concentration used (*μ*g/mL)		% Recovery ± SD^a^					
		RPP		^ 1^DD		MCR	
DCL	PAN	DCL	PAN	DCL	PAN	DCL	PAN
8	8	99.63 ± 0.61	100.36 ± 0.44	99.86 ± 0.87	100.24 ± 0.67	100.42 ± 0.47	99.95 ± 0.80
8	10	100.42 ± 0.74	100.55 ± 0.87	99.55 ± 0.58	101.02 ± 0.77	100.43 ± 0.55	100.55 ± 0.58
8	16	99.78 ± 0.96	100.19 ± 0.86	99.94 ± 0.79	100.39 ± 0.63	100.02 ± 0.74	100.71 ± 0.89
10	8	100.63 ± 0.73	99.48 ± 0.56	100.58 ± 0.74	100.30 ± 0.62	99.77 ± 0.67	99.72 ± 0.94
10	10	100.72 ± 0.89	99.92 ± 0.61	100.12 ± 0.71	100.41 ± 0.83	100.19 ± 0.62	99.90 ± 0.68
10	16	99.42 ± 0.76	100.49 ± 0.62	100.07 ± 0.98	99.83 ± 0.58	99.98 ± 0.76	99.77 ± 0.95
16	8	99.90 ± 0.69	99.76 ± 0.95	100.77 ± 0.97	99.63 ± 0.76	99.84 ± 0.50	99.64 ± 0.62
16	10	100.32 ± 0.57	100.46 ± 0.88	99.90 ± 0.68	99.86 ± 0.84	99.66 ± 0.61	100.48 ± 0.59

Mean		100.04	100.09	100.10	100.15	100.10	100.21
Mean RSD (%)		0.617	0.758	0.744	0.723	0.793	0.714

^a^Mean values of three replicates; RPP: ratio spectra peak to peak measurement method; ^1^DD: ratio derivative method; MCR: mean centering of ratio spectra method; % RSD: relative standard deviation.

**Table 4:** Determination of diclofenac and pantoprazole in DUFEX tablets by the proposed methods and the application of standard addition technique.

Claimed (mg)	Analysis of DUFEX tablets			Standard addition				
	% Recovery^a^ (% mean ± SD)			Taken (*μ*g/mL)	Added (*μ*g/mL)	% Recovery^a^ (% mean ± SD)		
	RPP	^ 1^DD	MCR			RPP	^ 1^DD	MCR
Diclofenac								
75	99.71 ± 1.04	100.60 ± 0.83	99.65 ± 1.04	7.5	6.0	100.16 ± 0.64	100.17 ± 1.28	99.68 ± 1.23
				7.5	7.5	100.23 ± 1.19	98.97 ± 0.83	99.25 ± 0.68
				7.5	8.0	99.75 ± 0.86	100.35 ± 0.86	99.84 ± 0.99
					Mean	100.05	99.83	99.59
					SD	0.899	0.990	0.968

Pantoprazole								
20	100.64 ± 0.57	100.35 ± 0.77	99.79 ± 0.89	2.0	1.6	100.81 ± 0.84	100.55 ± 1.21	99.22 ± 1.12
				2.0	2.0	100.21 ± 0.58	100.39 ± 0.83	99.45 ± 1.18
				2.0	2.4	99.48 ± 0.87	99.37 ± 0.84	100.40 ± 0.94
					Mean	100.17	100.10	99.69
					SD	0.767	0.960	1.078

^a^Mean of three replicate measurements; RPP: ratio spectra peak to peak measurement method; ^1^DD: ratio derivative method; MCR: mean centering of ratio spectra method; SD: standard deviation.

**Table 5:** Results of single factor ANOVA for comparison of proposed methods for the determination of diclofenac (DCL) and pantoprazole (PAN).

Drug	Source of variation	Degree(s) of freedom	Sum of squares	Mean square	*F*-value^a^	*P* value
DCL	Between columns	2	0.9445	0.4723	0.8877 (5.1433)	0.4595
	Within columns	6	3.1920	0.5320		
	Total	**8**	**4.1365**			

PAN	Between columns	2	0.0455	0.0227	0.9876 (5.1433)	0.4258
	Within columns	6	0.1381	0.0230		
	Total	**8**	**0.1836**			

^a^Values in parentheses indicate the critical value of *F*.
